# Benefits for Older People Engaged in Environmental Volunteering and Socializing Activities in City Parks: Preliminary Results of a Program in Italy

**DOI:** 10.3390/ijerph17113772

**Published:** 2020-05-26

**Authors:** Cristina Gagliardi, Karl Pillemer, Elena Gambella, Flavia Piccinini, Paolo Fabbietti

**Affiliations:** 1 Centre for Socio-Economic Research on Ageing, IRCCS INRCA—National Institute of Health & Science on Ageing, 60124 Ancona, Italy; e.gambella@inrca.it (E.G.); f.piccinini@inrca.it (F.P.); 2 Department of Human Development, Cornell University, Ithaca, NY 14850, USA; kap6@cornell.edu; 3 Laboratory of Geriatric Pharmacoepidemiology, IRCCS INRCA—National Institute of Health & Science on Ageing, 60124 Ancona, Italy; p.fabbietti@inrca.it

**Keywords:** older people, volunteering, physical activity, pro-environmental attitude, subjective well-being

## Abstract

The objective of this pilot study was to investigate the feasibility of an environmental volunteering program involving park restoration and social activities for older people. Older people face a series of challenges, ranging from social isolation and depression to a lack of exercise, as well as the difficult task of creating new meaning to deal with a decrease in vitality and new social relationships, interests, and commitments. From this perspective, this pilot study aimed to contribute to highlighting if or how these aspects can be positively correlated with organized activities associated with caring for public green spaces. A single-group, pre-test/post-test design was used, and baseline and post-participation measurements were recorded. The data were collected using a questionnaire aimed at evaluating variations in physical activity, positive and negative emotions, life satisfaction, and perceived social support. Through focus group interviews with participants at the baseline and after one year of participating in the program, the participants’ motivations and experienced effects were explored. The sample was composed of a group of 19 healthy people who joined the program. The quantitative results showed that the participants’ level of physical activity, subjective life satisfaction, and positive feelings were significantly increased at the follow-up. Positive correlations were found between increasing moderate physical activities, walking, and The Positive and Negative Affect Schedule (PANAS) positive feelings. A pro-environmental attitude of the elderly emerged from the qualitative data. The results, limitations, and improvements of the study are discussed.

## 1. Introduction

The strategy associated with promoting physical activity at all ages, recently promulgated by the WHO for the European Region for the years 2016–2025, has increasingly been recognized as a vehicle for maintaining the good health and well-being of the population [1]. Governments and interested parties should work to increase the levels of physical activity of all citizens, creating conditions to reduce sedentary behavior. Cities can act as WHO partners by adopting a national WHO Healthy Cities approach [2,3]. They can work toward increasing the quality of urban settings, enhancing local resilience, and promoting sustainable lifestyles through green spaces and other nature-based solutions. This approach implies the integration of the municipality and health authorities’ programmatic choices with other actors of the local community. From this perspective, the active contribution of older people in supporting community-based and voluntary activities is of critical importance [4].

Consistent with this framework, a program of civic environmentalism engaging older adults over 65, who are in good health and have a good level of mobility, was promoted by the Municipality of Ancona, Italy, in the year 2017. The municipality made use of the collaboration of “Associazione per i Diritti degli Anziani” (ADA), a voluntary association for the rights of the elderly, with a significant amount of experience in environmental volunteering, the local Health prevention office and IRCCS INRCA, a national research institute operating in the geriatric and gerontology fields. The program received an award for transferability and ease of implementation from the Italian National Network Healthy Cities of the WHO in 2018. A survey on the potential benefits in terms of well-being obtained by the participants at the end of the experience was included. This article reports the results.

### 1.1. Benefits of Environmental Volunteering for Health

In general, a recognized benefit of volunteering is helping people to believe that they can make a positive difference in the world and feel good about themselves [5]. The specific benefits of volunteering in environmental initiatives and natural areas are improvements in mental and physical health [6] and increases in physical activity [7] and self-esteem [8]. A growing body of studies has demonstrated that activities associated with environmental volunteering, like cleaning natural areas, gardening, and weeding, may provide several benefits, such as a positive mood and increased vitality [9], increased physical activity [10,11], and a reduction in the risk of falling [12]. A qualitative study reported health and wellbeing enhancement as one of the main benefits experienced by older volunteers engaged in a restoration program in urban parks [13].

Finally, gardening offers the possibility of admiring and being in settings of natural beauty, and the possibility of appreciating something aesthetically beautiful provides a tool for developing resistance and overcoming stress or unpleasant situations [14].

### 1.2. Benefits for Socialization

Experiencing nature with others facilitates communal and shared experiences. In particular, environmental volunteering is useful for counteracting the risk of social isolation of older persons by bringing together people in a meaningful activity in nature [15], thus providing them the chance to belong to a group and build social connections [16]. The sharing of experiences [17] and common community tasks [18] have been highlighted in the literature on the social benefits of environmental volunteering as activities that increase one’s sense of place ownership and community cohesion [16,19].

### 1.3. Social Identity and Pro-Environmental Attitude

Caring for a specific place represents a possible way of maintaining a social role, offering an opportunity to develop new knowledge and face new tasks (e.g., volunteering in gardening activities and educational sessions taught by experts) [13,19,20]. As a result, older volunteers may assume new identities, such as ‘caretakers, guardians and advocates’ of the people and places where they live, redefining their social roles in a positive and meaningful way [20,21]. In turn, by caring for places, volunteering can potentially lead to a greater concern for the environment and changes in people’s behavior towards more pro-environmental activities [15,21].

### 1.4. Study Objectives

The present study aimed to investigate the feasibility of a program of environmental volunteering and socializing activities in city parks for older people. We tested the implementation of a recruitment strategy, continuity of participation, and design of the study in order to identify and resolve as many potential problems as possible. The quantitative and qualitative data were collected to investigate the results in terms of the well-being and experienced outcomes of the participants after one year of the activities.

## 2. Materials and Methods

### 2.1. Recruitment Process and Program Characteristics

This civic environmental volunteering program was implemented in collaboration with the association for the rights of the elderly (ADA), which recruited and coordinated volunteers, as well as served as a presenter with IRCCS INRCA. In the recruitment phase, presentation meetings were organized at the local elderly associations and social centers, explaining the relevance of the project for the community as well as the benefits of being in the nature.

The program offered older participants the opportunity to experience outdoor activities carried out in two city parks through sustainable gardening practices. Activities in the parks were scheduled twice a week. Tasks were cleaning up less well-groomed areas, removing twigs along the trails, reporting potentially dangerous situations, and maintenance and repair work in damaged areas or equipment where it was possible. The ADA association held training meetings to provide the volunteers with the necessary skills to complete the tasks assigned, as well as necessary information in terms of safety. Moreover, in order to increase the motivation of potential participants, to encourage participation, and avoid doing too many repetitive things, a certain autonomy in planning new activities was given in accordance with the municipality. Support to the participants was guaranteed both for the implementation of the activities closely related to the care of the parks as well as for the organization of socializing activities responding to their needs and expectations, e.g., seminars on the adoption of a healthy lifestyle to be held in the parks. There were no formal inclusion or exclusion criteria. It was required to be able to carry out at least one of the proposed activities.

### 2.2. Data Collection and Research Design

For this pilot study, a single-group, pre-test/post-test design was used, and baseline and post-participation measurements were recorded. A descriptive survey (quantitative) and two focus group interviews (qualitative) were conducted to identify the benefits for well-being of environmental volunteering after one year of participation in the program. During this time none of the participants left the program. With the qualitative analyses, we were also able to explore the meaning and content of participants’ experiences regarding volunteering in nature. Ethical approval was not mandatory for this study, according to Italian law. Data anonymity and privacy were guaranteed through a consent letter, which was signed by the participants to allow for the processing of personal data.

### 2.3. Quantitative Survey

The data were collected using a questionnaire, which aimed to evaluate the variation in the following variables: The Physical Activity Scale for the Elderly (PASE), Positive and Negative Affect Schedule (PANAS), Life Satisfaction, and Lubben Social Network Scale (LSNS). All the research tools were validated for use in older people. Trained research assistants (a psychologist and social researcher) managed the protocol administration, which required approximately 20 min per participant and provided support to participants who had difficulties in completing the questionnaire independently. The in-person administration allowed the interviewers to personally interact with each participant, clarifying questions and reviewing answers when necessary. The questionnaires were administered in May 2017 and after one year, in May 2018. The Physical Activity Scale for the Elderly (PASE) was administered as a subjective estimate of the physical activity of the participants [22]. The 13-item scale assesses the amount of time spent in each “recreational leisure activity” (6 items) and “household activity” (7 items) carried out by the participants over seven days, prior to filling out the questionnaire. For each activity, the frequency (number of times per week) and duration (in minutes) were assessed. The Positive and Negative Affect Schedule (PANAS) [23], a self-reporting questionnaire, was used to assess the frequency (from never to always) of the positive and negative emotions on a 5-point scale. The questionnaire consists of 20 items, which are divided in two scales, each with 10 items: 10 adjectives for the positive affect scale (PA) and 10 for the negative affect scale (NA). The factorial structure of the test assumes two factors, which correspond to the two subscales (PA and NA), which are independent from each other. The reliability and validity of PANAS have previously been tested in aged people. The Positive and Negative Affect Schedule (PANAS) has been validated in Italy [24]. A single-item Life Satisfaction Measure was used to assess how satisfied participants were with their lives on an 11-step Likert scale [25]. The Lubben scale consists of seven items that assess the closeness and frequency of contacts with relatives and friends. Each subscale (family subscale and friends subscale) was comprised of three items that referred to the number of network members providing regular (at least monthly) contact; the frequency of contacts with the member, with whom the member felt sufficiently “close”; and the number of network members, who “can be called on for help and can be confided in about private matters”. Finally, one item investigates the general perception of social support: “When making an important decision do you have someone to talk to?” The participants responded on a 6-point scale, from 0 (none) to 5 (nine or more) to assess the size of the network, from 0 (less than once a month) to 5 (every day) to assess the frequency of contacts, and finally, from 0 (never) to 5 (always) to assess the last item.

### 2.4. Qualitative Interviews

As older people’s participation in environmental volunteering is a relatively new topic, two focus groups were conducted to gather information from the participants about their motivation to participate in the project at the baseline and their experienced outcomes at the end [26]. The participants were requested to sign consent forms, before the focus group started, following a brief introduction. The principal investigator facilitated the discussions, and a research assistant observed and took notes. At the baseline, the focus group discussion focused on the participants’ motivation to participate in the project, and their expectations and opinions were used as cues for discussion. The follow-up discussion focused on the participants’ subjective experiences, and it was conducted with the following themes: experienced well-being, engagement, opinions about learning and working in the environment, and civic volunteering. Each interview was audiotaped, and field notes were taken.

### 2.5. Statistical Analysis

The data were expressed by the mean and standard deviation for the continuous variables and by the number of cases and percentage for the categorical ones. Comparisons between the baseline and follow-up were carried out using the paired sample Student’s *t*-test for continuous variables and the chi-square test for categorical variables. “Delta” variables, expressing the variations between the follow-up and baseline, were created in order to examine the associations by means of Pearson’s correlation coefficient. A *p* value < 0.05 was considered to be statistically significant. Statistical analysis was conducted using SPSS for Win V24.0 (SPSS Inc., Chicago, IL, USA).

## 3. Results

### 3.1. Quantitative Results

The sample was composed of 11 men and eight women (Table 1). The mean age was 75.7. The majority of participants (68.4%) had a lower level of education (primary and lower secondary education) and were married/cohabiting (84.2%). Walking was performed by 84.2% of the sample, light sport by the 47.4%, moderate sport by the 26.3%, and strenuous sport, lifting weights, and pushups by 10.5%.

More than 73% performed light or moderate sport, while 21% performed strenuous sports and weightlifting. Concerning health, 15.8% of the sample declared that they had cardio-vascular disease, 15.8% had cancer, and 26.3 had osteoporosis.

Comparisons of the variables between the baseline and follow-up (Table 2) that exhibited significant differences are shown (*p* < 0.05). Some individual items of the PASE presented a significant variation: weekly engagement in gardening activities and caring for another person increased by 30% and 20%, respectively; the mean weekly hours of volunteering performed by the participants increased from 2.2 to 7.7; and the mean level of physical commitment to carrying out the volunteering activities increased from 1.1 to 2.8, with a scale of 1–4. Finally, the PASE score, categorized on the basis of the 50th percentile, which was equal to 195.18, showed a positive variation. The Lubben scale did not show significant variations, except for a significant increase in the frequency of interaction with relatives. Subjective satisfaction with life increased from 7.2 to 8. The PANAS scale was significantly raised in terms of feeling interested, strong, enthusiastic, proud, alert, determined, attentive, and active. Accordingly, the PANAS positive affect increased from 31.8 to 37.6. Conversely, feeling distressed decreased from 2.7 to 2.3.

Table 3 shows that increasing walking was correlated with increasing PANAS positive feelings, such as strong, proud, determined, alert, and active, and with the general positive affect score. Likewise, an increase in moderate physical activity was correlated with increasing positive PANAS affect variables, such as feeling interested, strong, and enthusiastic, and with the general positive affect score. Strenuous physical activity was negatively correlated with the enthusiasm score. A positive variation in making decisions was positively correlated with the positive feelings of being interested and enthusiastic.

### 3.2. Qualitative Results

Only some of those who completed the questionnaire agreed to participate in the focus groups. Consequently, 13 older people participated in the baseline focus group and 10 in the follow-up. The qualitative data included participants’ responses to open questions. The analysis was undertaken manually in light of the small amount of data. The transcripts were examined using constant comparison and thematic analysis [26]. Meaning units were identified and extracted from the transcriptions through text shortening, maintaining their meaning. Similar meaning units were then coded, and categories were identified (“Categories” in Table 4). Common themes, reflecting the contents of each category, were finally developed (“Themes” in Table 4). The inter-coder reliability was verified at each stage by checking the coded data and discarding any data that had not been mutually coded or agreed.

Concerning the participants’ motivation to participate in the program (Table 4), four themes were drawn from the data analysis: “project aims and characteristics”, “socialization”, “social identity”, and “benefits of nature.” The project characteristics encompass sub-themes that referred to the aims of the project, namely, environmental restoration and civic engagement, stability, closeness, and freedom to participate. Socialization refers to the desire to have new relationships and the need to belong to a peer group, while social identity refers to the need for a social role, like being a resource and having a purpose. Finally, the benefits of nature encompass staying in nature, exercising, and relaxing.

Positive outcomes on the participants’ emotional and social well-being were described by the participants after the year-long program (Table 5). In particular, three themes were drawn: “well-being”, “agency”, and “social support.” Well-being encompasses mental and physical health, while agency refers to the possibility of planning activities, youth education, environmental, and civic engagement. Social support refers to a positive group and family interactions.

## 4. Discussion

This study was conducted to examine the feasibility of a civic environmental volunteering program for older people. Older people face a series of challenges, ranging from social isolation and depression to a lack of exercise, as well as the difficult task of creating new meanings to deal with a decrease in vitality and new social relationships, interests, and commitments [27]. From this perspective, the aim was to investigate whether and how these aspects can be positively correlated with organized activities aimed to care for public green spaces. After one year of experience in the environmental restoration of parks, together with socializing activities, positive variations were found in the selected variables. Concerning physical activity, the qualitative results highlighted that the need for going outdoors and getting exercise were among the reasons to participate. At the end of the year during which the participants engaged in the activities in the parks, the quantitative results showed that the participants’ level of physical activity increased significantly, though the sample was already healthy and physically active at the baseline, presenting high PASE scores (the lowest standing being 82). For this reason, while previous literature [28] established four categories of physical activity, from an inactivity condition (PASE < 42) to an intense physical activity (PASE > 146), our selected cut-off was much higher than that in the cited literature (50th percentile, equal to 195.18.

Following Diener [29], subjective well-being was conceptualized by means of cognitive and affective components, the first based on a life satisfaction construct, which represents an account of how a respondent evaluates his or her life as a whole, and the second assessed through positive and negative emotions, which reflect the amount of pleasant and unpleasant feelings that people experience. Consistent with the national data showing that older people are less satisfied than the whole population [30], the mean value of subjective satisfaction for the whole sample was equal to 7.2, which is lower than the national mean value of 7.6 [31].

An important result was that, after the experience, the mean value increased to 8.0, and pleasant feelings measured by the PANAS scale showed a significant increase. On the contrary, the feeling of distress significantly decreased from 2.7 to 2.3. This result supports that mental health benefits are one of the main outcomes of volunteering in the nature, as other previous studies have observed [32]. Positive correlations were found between increasing walking and PANAS positive affect, namely, feeling strong, proud, determined, alert, and active. Likewise, an increase in moderate physical activities was correlated with increasing positive PANAS feelings. This result provides additional support to previous literature by highlighting the positive outcomes of outdoor exercise, as well as of being in nature, for mood and increased vigor and energy [33].

While the Lubben scale was used to measure the level of social support experienced by the participants, it did not show a significant variation, except for increasing interactions with relatives. This result may be due to the sharing of experiences within a family, which was also reported by the participants in the qualitative results, thus suggesting a positive feedback in network relationships.

The qualitative findings highlighted that there were several positive outcomes of the experience in terms of group interactions. Indeed, some aspects of the experience, such as working on a common task and learning new skills, contributed to the creation of strong relationships between participants.

Among the activities that participants planned independently, is worth mentioning the design and construction of a rose garden, which was not included in the initial project but resulted from their initiative.

This aspect adds value to the civic volunteering program model that aimed to meet the needs of older people, avoiding assigning too repetitive tasks, and allowing them to show independence. These results add more evidence to the statement of Lewis [34], who pointed out that for elderly people who have decreased personal and social commitments, taking care of plants and flowers can represent a valid “substitute for interest”, guaranteeing an opportunity for the prospect of tomorrow. The noteworthy level of engagement of the participants finds confirmation in the high attendance in scheduled activities, which led to an increase in the hours dedicated to volunteering (from 2.2 to 7.7).

Moreover, the qualitative data reported that the personal gain through the social bonds formed among the members was not the only aspect that participants attributed to the experience; good relationships and support established at the community level were also valued. Indeed, contact with the visitors of the parks was highly appreciated, and the participants said that they felt proud to be a role model for visitors and that the impact of their influence on visitors was acknowledged.

A pro-environmental attitude of the elderly emerged from the qualitative data. The participants declared that the reasons for attending the project were environmental volunteering and the concreteness of the activities aimed at maintaining the park. Moreover, the discussion on this topic highlighted environmental benefits as a motivating factor. The sense of community was attributed by participants to the environmental nature of the project.

Among the benefits of the experience, the participants also mentioned the opportunity to plan activities together in the environment and being involved in goal-oriented tasks, for instance, designing the rose garden, because visitors could enjoy the participants’ enhancement of it. In this respect, previous studies highlighted how agency, that is, the capacity to exert power through environmental actions, ref. [35] fulfills a desire to contribute to a collective future that outlives oneself [36]. Thus, we can say that designing the rose garden fulfilled the participants’ desire to nurture the environment and create a better future [37].

Finally, encouraging a pro-environmental attitude among young people frequenting the parks was reported as an aspect of the group identity, as well as a way of increasing civic engagement among the youngest, confirming that having new roles as gardeners may give people a positive identity and something to share with people outside the group [38].

In conclusion, elderly people may derive several benefits from environmental volunteering, the experience of which provides them with physical and social activity, as well as a sense of purpose at a time when their social roles are changing. This is extremely interesting, considering that having a purpose or meaning in life turns out to be a protective factor against mortality and frailty [39,40].

## 5. Limitation of the Study

The small sample size (*n* = 19) of this pilot study allowed for some statistical analysis and preliminary results useful for an evaluation of feasibility and proof of concept of conducting a before-and-after study in future research. Our preliminary results suggest that this kind of program could potentially be a valid option for obtaining benefits in terms of physical, mental and social health, but it must be further tested in a healthy old population representative sample.

## 6. Future Research

Two substantial issues should be addressed: first, expanding the publicity of the initiative and improving some organizational aspects; and second, assessing the process of auto-selection due to the voluntary nature of the program. For the pilot study we have limited the recruitment phase to associations and centers for the elderly. Considering that being unware of opportunities in the community is one of the barriers to participation in environmental volunteering programs [41], a future study should include a recruitment strategy involving all advertising possibilities that may be suitable for reaching a larger audience of the elderly population.

From an organizational point of view, one issue with this study concerned the limited number of city parks in which the program was conducted in the pilot phase, which favored the participation of those who lived nearby and discouraged the inhabitants of other neighborhoods, who complained about the impossibility of constantly going to parks not close to their home. This issue should be taken into consideration for planning future recruitment, for example increasing the number of parks in which the program is conducted and providing a dedicated budget for a public transport plan.

Another issue is that a healthy group resulted from the auto-selection of participants. We argue that the program was attractive for healthy older people in the recruitment phase. This aspect of the recruitment inhered not only in the voluntary nature of the adhesions, but above all in the nature of the program particularly attractive/suitable for people in good health, and it therefore deserves to be considered in future planning.

## 7. Conclusions

This pilot study was useful for identifying and evaluating issues associated with recruiting a sample of older volunteers for a new civic program to test the dataset and the study protocol, as well as for identifying suggestions for future research. Participating in an environmental volunteering program associated with environmental restoration and social activities in city parks is associated with physical, mental, and social benefits for older people in good health.

## Figures and Tables

**Table 1:** Characteristics of the sample.

Variables	Baseline
	(*n* = 19)
Female, *n* (%)	8 (42.1)
Age, years	75.7 ± 5.1
Primary Education	4 (21.1)
Lower Secondary Education	8 (42.1)
Upper Secondary Education	5 (26.3)
Tertiary Education	2 (10.5)
Married/Cohabiting	16 (84.2)
Single	0 (0.0)
Widowed	3 (15.8)
Reading, watching television, doing handcrafts, *n* (%)	19 (100.0)
Taking a walk, walking the dog, walking to work, walking in a mall, *n* (%)	16 (84.2)
Light sport (e.g., low-impact exercise, fishing, etc.), *n* (%)	9 (47.4)
Moderate sport (e.g., dancing, hunting, doubles tennis, etc.), *n* (%)	5 (26.3)
Strenuous sport (marathon, cycling, singles tennis, aerobic dance, etc.), *n* (%)	2 (10.5)
Lifting weights, pushups, *n* (%)	2 (10.5)
Chronic nonspecific lung disease (asthma, bronchitis, emphysema), *n* (%)	0 (0.0)
Cardio-vascular disease, *n* (%)	3 (15.8)
Peripheral Arterial Disease (arteriosclerosis), *n* (%)	0 (0.0)
Diabetes mellitus, *n* (%)	0 (0.0)
Stroke, *n* (%)	0 (0.0)
Cancer, *n* (%)	3 (15.8)
Osteoporosis, *n* (%)	5 (26.3)

**Table 2:** Pre- and post-program significant variables.

Variables	Baseline	Follow-up (*n* = 19)	*p*-Value
	(*n* = 19)		
PASE *: Housework activity over the past 7 days: outdoor gardening, *n* (%)	13 (68.0)	18 (95.0)	0.021
PASE: Housework activity over the past 7 days: caring for another person, *n* (%)	12 (63.0)	16 (84.0)	0.042
PASE: Paid/voluntary work over the past 7 days: hours	2.2 (2.8)	7.7 (7.3)	0.009
PASE: Amount of physical activity required by job/volunteering	1.1 ± 1.5	2.8 ± 0.8	<0.001
PASE < 195.18	8 (42.1)	6 (31.6)	0.013
Lubben scale: frequency of interaction with relatives	3.3 ± 1.7	4.3 ± 1.2	0.014
Subjective satisfaction with life	7.2 ± 1.4	8.0 ± 1.1	0.016
PANAS **: interested	3.7 (0.8)	4.2 (0.7)	0.016
PANAS: distressed	2.8 (0.7)	2.3 (0.8)	0.025
PANAS: strong	2.7 (1.2)	3.6 (1)	0.031
PANAS: enthusiastic	3.1 (1.2)	4 (0.8)	0.011
PANAS: proud	2.7 (1.1)	3.4 (0.8)	0.038
PANAS: alert	3.3 (0.9)	3.9 (1)	0.014
PANAS: determined	3.4 (0.8)	4 (0.8)	0.002
PANAS: attentive	3.6 (1)	4.2 (0.6)	0.030
PANAS: active	4.2 (0.8)	4.6 (0.5)	0.025
PANAS POSITIVE	31.8 ± 6.3	37.6 ± 3.7	0.003
PANAS NEGATIVE	24.0 ± 4.6	21.7 ± 4.7	0.083

* The Physical Activity Scale for the Elderly, ** The Positive and Negative Affect Schedule.

**Table 3:** Delta Pearson’s correlations.

Pearson’s Correlations (*p*)								
	Interested	Strong	Enthusiastic	Proud	Determined	Alert	Active	PANAS Positive Affect
Walking (min)	−0.024 (0.923)	0.553 (0.014)	0.266 (0.271)	0.592 (0.008)	0.534 (0.019)	0.776 (0.009)	0.520 (0.022)	0.659 (0.002)
Moderate physical activity (min)	0.488 (0.034)	0.622 (0.004)	0.673 (0.002)	0.386 (0.103)	0.386 (0.103)	0.206 (0.397)	0.036 (0.883)	0.564 (0.012)
Strenuous physical activity (min)	−0.203 (0.405)	−0.412 (0.079)	−0.539 (0.017)	−0.204 (0.402)	−0.095 (0.699)	0.161 (0.510)	0.238 (0.326)	−0.385 (0.103)

**Table 4:** Motivation to participate in the program.

Codes	Categories	Themes
Benefits for the park	Environmental engagement	Project aims and characteristics
Environmental volunteering		
Concrete tasks		
Desire to help others	Civic engagement	
Benefiting many others		
Inter-institutional cooperation	Stability	
Project value		
Dissemination		
Closeness	Mobility	
Neighborhood		
Improvisation	Freedom	
Not binding		
Making new friends	New relationships	Socialization
Need for human contact		
Being part of a group	Group	
Comparison with peers		
Elder as a resource	Being a resource	Social identity
Having a purpose	Purpose	
Motivation to go out		
Doing new things		
Benefits of nature	Nature	Benefits of nature
Spending time in the park		
Staying in nature		
Walking	Physical activity	
Exercising		
Getting one’s mind off of things	Relaxing	

**Table 5:** Experienced outcomes.

Codes	Categories	Themes
Positivity	Mental health	Well-being
Calmness		
Self-esteem		
Overcoming laziness	Keeping active	
Being active		
Goal-oriented tasks	Planning	Agency
Planning activities together		
Encouraging a pro-environmental attitude Among young people	Youth education	
Providing an example for young people		
Usefulness to others	Environmental and civic engagement	
Cleanliness of the park		
Being thanked		
Seniors as positive models		
Creating a tight-knit group	Group interaction	Social support
Learning from each other		
Peer support		
Information exchange		
Telling family about it	Consequences on the personal/familial environment	
Sharing my experiences with family		
Children encouraging me to continue		
Family noticing improvements in my mood		
